# Efficacy of Baduanjin exercise for rehabilitation after COVID-19: A protocol for systematic review and meta-analysis

**DOI:** 10.1097/MD.0000000000026366

**Published:** 2021-06-18

**Authors:** Jiao Rong, Jing Li, Fushi Jing, Yonghui Ren, Yunpeng Xiao, Qi Pan, Mengtian Li, Yueming Lv, Jing Zhang, Fujie Jing

**Affiliations:** a School of Acupuncture-Tuina, Shandong University of Traditional Chinese Medicine, Jinan; b Acupuncture and Massage Department, Weifang Hospital of Traditional Chinese Medicine, Weifang; c Department of Rehabilitation, The People's Hospital of Juxian; d Department of Rehabilitation, The Second Affiliated Hospital of Shandong University of Traditional Chinese Medicine, Jinan, Shandong, China.

**Keywords:** Baduanjin, COVID-19, meta-analysis, rehabilitation, systematic review

## Abstract

**Background:**

The study aims to evaluate the effectiveness and safety of Baduanjin exercise for rehabilitation after COVID-19.

**Methods:**

The following electronic databases will be searched from establishment to Jan 2021: Cochrane Library, MEDLINE, EMBASE, Web of Science, Springer, World Health Organization International Clinical Trials Registry Platform, China National Knowledge Infrastructure, Wan-fang database, Chinese Scientific Journal Database, Chinese Biomedical Literature Databases, and other databases, All published randomized controlled trials about this topic will be included. Two independent researchers will operate article retrieval, duplication removing, screening, quality evaluation, and data analyses by Review Manager (V.5.3.5). Meta-analyses, subgroup analysis, and/or descriptive analysis will be performed based on the included data conditions.

**Results:**

The results of this study will provide a combination of high-quality evidence for researchers in the current field of COVID-19 treatment and rehabilitation.

**Conclusion:**

The conclusion of this study will provide the evidence of whether Baduanjin is an effective and safe intervention for rehabilitation after COVID-19.

**PROSPERO registration number:**

CRD42020181078.

## 1 Introduction

### 1.1 Description of the condition

Coronavirus disease 2019 (COVID-19) is a respiratory illness that can spread from person to person. Its infectivity is wide and pathogenicity is strong.^[1]^ As of May 14, 2021, >1614 million people have been infected worldwide. This is a terrible figure that poses a huge challenge to global health and brings huge losses to the global economy. Currently, the sequelae of COVID-19 patients are rarely reported, but the impact on people's quality of life is inevitable, especially in people with COVID-19 sequelae. COVID-19, as a sudden outbreak, pandemic infectious disease, causes an emotional response of extreme fear and uncertainty in the public, which often leads to negative social behavior and may involve public mental health issues such as anxiety, depression, insomnia, aggression, depression, and hysteria.^[2,3]^ Given the huge physical and psychological damage COVID-19 has caused to human beings and the lack of good therapeutic interventions, a simple, reliable, and feasible treatment is urgently needed to improve the physical and mental health of the public.

### 1.2 Descriptiom and function of intervention

Baduanjin, one of the most common forms of Qigong exercise, dates back to the Chinese Song Dynasty (10^th^–13^th^ centuryA.D). It is composed of 8 simple independent movements and characterized by slow movements, mental concentration and meditation, regulated breathing to achieve a harmonious flow of Qi in the body.^[4]^ In recent years, due to its effectiveness for keeping fit, ease in learning, and economy of exercising time, Baduanjin has become popular worldwide as a promising low-intensity, physical and mental exercise.^[5]^ It is beneficial to the quality of life, sleep quality, balance, grip strength, trunk flexibility, systolic and diastolic blood pressure, and resting heart rate.^[6]^ It also plays a role in reducing depression and anxiety symptoms in patients with psychosomatic illnesses.^[7]^ It can improve cognitive function in different age groups and different clinical groups, as well as psychological and physiological parameters.^[8]^ It has the characteristics of good analgesic effect, no side effects, convenient operation, low economic burden, and more conducive to promoting the rehabilitation of patients.

### 1.3 Why the review is important

The Covid-19 pandemic has caused untold disruption and enhanced mortality rates around the world.^[9]^ As a new infectious disease, there is no specific drug at present.^[10]^ As a treasure of Chinese national culture, body-building Qigong, mainly based on Baduanjin exercise (BDJE), has been recommended by many medical programs or experts for the treatment and rehabilitation of COVID-19.^[11–13]^ Studies have shown it can boost immunity, improve lung function, and relieve symptoms of depression.^[14–20]^ However, the evidence was still limited based on nonstandard measurement, nonuniformed outcomes, subjectivity judgment, and other factors. However, no related review or protocol has been published. To evaluate the efficacy and safety of BDJE for rehabilitation after COVID-19, it is necessary to conduct evidence-based review. So, this review is urgently needed to accomplish.

## 2 Methods

This systematic review protocol has been registered in the PROSPERO. The registration number: CRD42020181078. All steps of this systematic review will be performed according to the Cochrane Handbook (5.2.0).

### 2.1 Selection criteria

#### 2.1.1 Types of studies

Randomized controlled trials (RCTs) and blinded research will be included. Published clinical trials that reported the efficacy on Baduanjin for rehabilitation after COVID-19 will be included. RCTs that involve at least 1 Baduanjin related treatment to COVID-19, and 1 control treatment (or blank treatment) will be included. As there is a risk of interference with the outcome, non-RCTs will be excluded. Observational, cohort, case–control, case series, qualitative and laboratory studies, and uncontrolled trials will be excluded.

#### 2.1.2 Types of patients

Patients who were diagnosed as COVID-19 will be included, without limits on sex, age, race, nationality, and medical units; all children <18 years of age will be included in the study.

#### 2.1.3 Types of interventions and comparisons

Interventions can be any type of Baduanjin. Multiple control interventions will be included: no treatment, placebo, and other interventions (eg, acupuncture, moxibustion, massage, cupping therapy, drugs, and physical interventions). If its interventions and comparisons both contain Baduanjin, the study will be excluded. Interventions of Baduanjin combined with other therapies will be included, only if these combinations are compared to the other therapies semplice.

#### 2.1.4 Types of outcomes

Primary outcomes will include mortality rate, cure rate, chest CT scans and nucleic acid detection of respiratory samples. Secondary outcomes will include improvement of Disappearance time of fever and cough, serum level of tumor necrosis factor-α and interleukin-6, side effects, and so on.

### 2.2 Search methods for identification of studies

#### 2.2.1 Electronic searches

The following electronic databases will be searched from establishment to Jan 2021: Cochrane Library, MEDLINE, EMBASE, Web of Science, Springer, World Health Organization International Clinical Trials Registry Platform, China National Knowledge Infrastructure, Wan-fang database, Chinese Scientific Journal Database, Chinese Biomedical Literature Databases, and other databases. All published RCTs about this topic will be included. Exemplary search strategy of MEDLINE is listed in Table 1, terms are conform to medical subject heading (MeSH). According to the difference of databases, keywords may combine with free words and comprehensive search will be performed.

**Table 1:** MEDLINE search strategy.

#1 MeSH Major Topic:COVID-19
#2 MeSH Major Topic: Coronavirus disease 2019
#3 MeSH Major Topic: 2019 novel coronavirus disease
#4 MeSH Major Topic: 2019-nCoV disease
#5 MeSH Major Topic: 2019 novel coronavirus infection
#6 MeSH Major Topic: SARS-CoV-2
#7 MeSH Major: Baduanjin exercise
#8 MeSH Major: Baduanjin
#9 MeSH Major: BDJE
#10 MeSH Major: BDJ
#11 MeSH Major: Qigong
#12 MeSH Major: eight section brocades
#13 #1 or #2 or #3 or #4 or #5 or #6
#14 #7 or #8 or #9 or #10 or #11 or #12
#15 #13 and #14

### 2.3 Data collection and analysis

#### 2.3.1 Selection of studies

**Figure 1:**
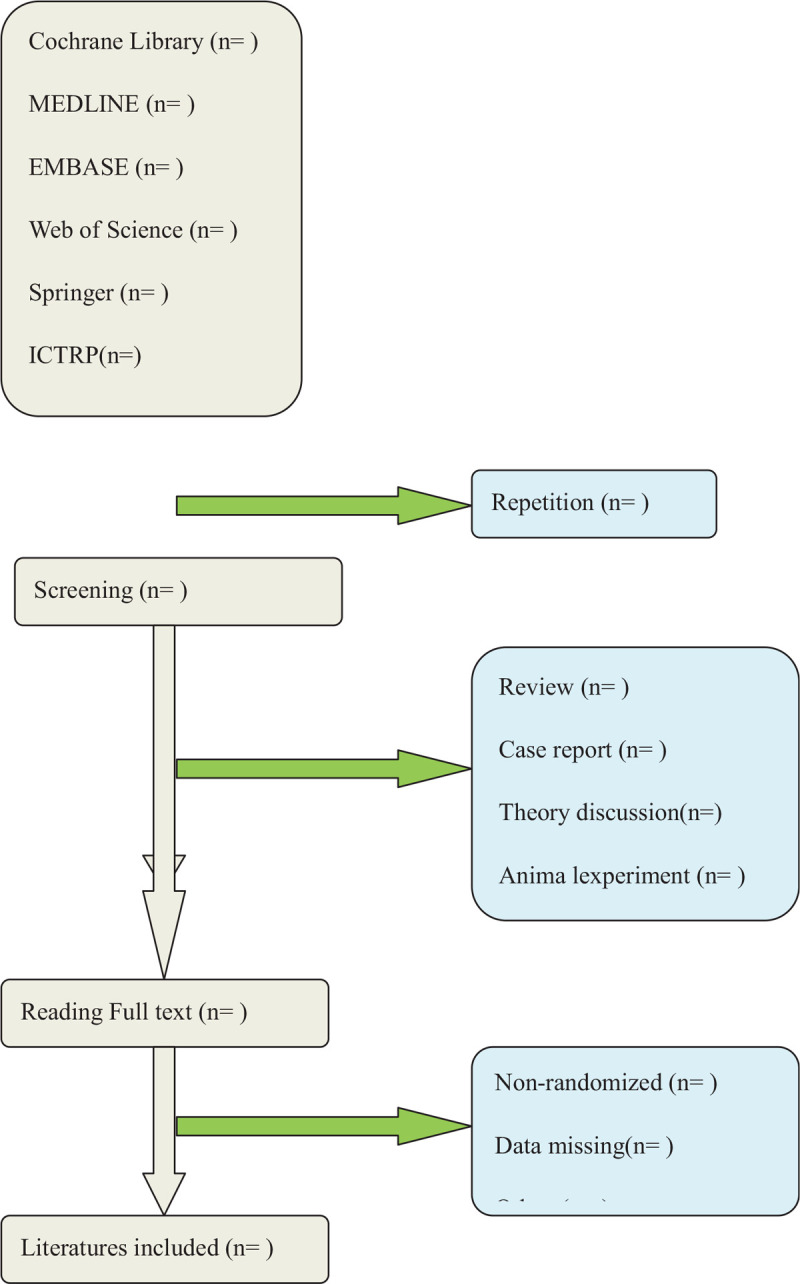
Flow diagram of studies identified.

Two authors (JL and JR) will select clinical trials depending on inclusion criteria. After the title and abstract are screened, literatures that are not related and do not meet the criteria will be excluded. Screening operation will flow the diagram of Figure 1. If the full literatures are unable to obtain or related data are incomplete, we will contact the corresponding author. Third-party experts will be consulted to determine the selection divergence.

#### 2.3.2 Assessment and quality of included studies

Two authors (JL and FSJ) will evaluate quality of included articles and assess the risk of bias based on Cochrane Handbook 5.2.0. Quality assessment of included studies contains randomized method, allocation concealment, blinding of participants and personnel, blinding of outcome assessment, completeness of outcome data, and selective reporting. Divergence of evaluation will also consult third-party experts.

#### 2.3.3 Data extraction

The authors (YPX and JR) plan to extract the data from the articles selected for inclusion, and to resolve differences in opinion through discussion with experts. Data will be recorded onto an electronic form, including categories for basic information about the studies (numbing, the first author's last name and the year the study was published, and the contact information for the corresponding author), the sample sizes and grouping methods used, participant characteristics including age and sex, expressed as mean additions and subtractions above and below standard deviation and the percentages, and details of the intervention methods involved, including treatment time, the selection of acupoints, treatment efficacy, treatment cycles, side effects, and follow-up.

#### 2.3.4 Measures of treatment effect

Two authors (MTL and QP) will perform analysis independently and then cross-check treatment effect with Review Manager 5.3.5. Dichotomous data will be presented by risk ratio with 95% confidence intervals. Continuous data will be presented by mean difference or standard mean difference with 95% confidence interval. Other binary data will be changed into the risk ratio form for analysis.

#### 2.3.5 Dealing with missing data

As there is possibility of missing data in literatures, we will contact the corresponding authors by email or other contacts. If the missing data are unavailable, we will analyze the existing data that are supposed as random missing.

#### 2.3.6 Assessment of heterogeneity

The heterogeneity of studies will be evaluated by *Q* test and *I*^2^ statistic with RevMan5.3.5. The following criteria will be used: *I*^*2*^ <50% will be deemed as low heterogeneity; *I*^*2*^ between 50% and 75% will be considered as moderate heterogeneity; *I*^*2*^ > 75% will be considered as high heterogeneity.

#### 2.3.7 Assessment of reporting bias

Publication bias and other reporting biases will be assessed by creating funnel plots. Symmetric funnel plots indicate low risk of bias, whereas dissymmetry ones may indicate high risk.

#### 2.3.8 Data synthesis

A meta-analysis or descriptive analysis will be performed, based on the intervention methods, the measurement methods, and heterogeneity levels, among others. If clinical and methodological heterogeneity are low, the fixed-effect model will be applied by merger analysis; the random-effects model will be applied by merger analysis when heterogeneity indicates a moderate level. If, however, a significant level of heterogeneity is found, a descriptive analysis will be performed instead.

#### 2.3.9 Subgroup analysis

Subgroup analysis will be performed based on the findings from the data synthesis, and if the heterogeneity is found to have been caused by particular features of the included studies (eg, the intervention methods [type, time, and cycle] and the measurement methods used in the clinical trials), subgroup analysis will be conducted relevant to these categories.

## 3 Discussion

As the number of people cured of COVID-19 increases, a large number of people will enter the recovery phase. People at this stage are often accompanied by a series of symptoms such as anxiety and insomnia. Baduanjin is widely used in patients recovering from COVID-19 in China due to its simplicity, convenience and low cost. Recently, there have been more and more clinical reports on the involvement of BDJE in COVID-19 treatment and rehabilitation, but high-quality trail is still insufficient. This review will begin when necessary trails are meeting. To give compelling evidence and better guide in clinic practice, all actions of this review will be performed according to Cochrane Handbook 5.2.0.

## Author contributions

**Conceptualization:** Jiao Rong, Jing Li, Fushi Jing.

**Data curation:** Jing Zhang, Yonghui Ren, Yunpeng Xiao.

**Investigation:** Qi Pan, Mengtian Li, Yueming Lv.

**Methodology:** Jiao Rong, Jing Zhang.

**Supervision**: Jing Li, Fushi Jing

**Validation:** Fujie Jing, Jing Zhang.

**Visualization:** Jiao Rong.

**Writing – original draft:** Jiao Rong, Jing Li.

## References

[1] Chen Y Ke H Qiu C. Current Situation and Research Progress of Novel Coronavirus. Laboratory Medicine and Clinical Practice 2021;18:131–3. +141.

[2] Shigemura J Ursano R Morganstein J. neurosciences c. Public responses to the novel 2019 coronavirus (2019-nCoV) in Japan: Mental health consequences and target populations. Pschyiatry Clin Neurosci 2020;74:281–2. 10.1111/pcn.12988 PMC7168047 32034840

[3] Ma Q Yang Z Zhu F. The effect of Baduanjin exercise on the quality of life in patients recovering from COVID-19: a protocol for systematic review and meta-analysis. Medicine (Baltimore) 2020;99:e22229. 32925800 10.1097/MD.0000000000022229 PMC7489728

[4] Zou L SasaKi J Wang H. A systematic review and meta-analysis Baduanjin Qigong for health benefits: randomized controlled trials. Evid Based Complement Alternat Med 2017;2017:4548706. 28367223 10.1155/2017/4548706 PMC5359459

[5] Zhou J Yu Y Cao B. Characteristic of clinical studies on Baduanjin during 2000-2019: a comprehensive review. Evid Based Complement Alternat Med 2020;2020:4783915. 33149753 10.1155/2020/4783915 PMC7603575

[6] Zou L SasaKi JE Wang H. A systematic review and meta-analysis Baduanjin qigong for health benefits: randomized controlled trials. Evid Based Complement Alternat Med 2017;2017:4548706. 28367223 10.1155/2017/4548706 PMC5359459

[7] Zou L Yeung A Quan X. Mindfulness-based Baduanjin exercise for depression and anxiety in people with physical or mental illnesses: a systematic review and meta-analysis. Int J Environ Res Public Health 2018;15:321. 10.3390/ijerph15020321 PMC5858390 29439556

[8] Zou L Pan Z Yeung A. A review study on the beneficial effects of Baduanjin. J Alternat Complementary Med 2018;24:324–35. 10.1089/acm.2017.0241 29227709

[9] Tang J Bahnfleth W Bluyssen P. Dismantling myths on the airborne transmission of severe acute respiratory syndrome coronavirus (SARS-CoV-2). J Hosp Infect 2021;doi:10.1016/J.JHIN.2020.12.022. 10.1016/j.jhin.2020.12.022 PMC7805396 33453351

[10] Cai Q Jing C Zhang X. Discussion on the role of fitness qigong in prevention of disease and the idea of prevention and treatment of COVID-19. Journal of Guangzhou University of Chinese Medicine 2020;37:1602–6.

[11] Zhang B Wang Q Gu X. Covid-19 TCM Diagnosis and Treatment Manual. China Press of Traditional Chinese Medicine 2020;25–6.

[12] Li J Zhang H. Covid-19 Expert Consensus on Chinese Medicine Rehabilitation (First Edition). Journal of traditional Chinese medicine 2020;35:681–8.

[13] Feng C Cui H Yu H. Covid-19 Expert Guidance on Comprehensive Traditional Chinese Medicine Intervention Program in Convalescence (Draft). Beijing Traditional Chinese Medicine 2020;39:102–4.

[14] Yang K Chang W Chuang H. Increased complement factor H with decreased factor B determined by proteomic differential displays as a biomarker of tai chi chuan exercise 2010;56:127–31. 10.1373/clinchem.2009.126615 19884489

[15] Cao Yun. Effects of body-building Qigong Baduanjin on HbA1c and immune function in patients with type 2 diabetes. Journal of Guangzhou Institute of Physical Education 2015;35:97–9.

[16] Jiqiang L liu N Yun X. Systematic evaluation of a stable randomized controlled trial of fitness qigong in the treatment of chronic obstructive pulmonary disease. Journal of Liaoning University of Traditional Chinese Medicine 2018;20:05–9.

[17] Liu SJ Ren Z Wang L. Mind-body (baduanjin) exercise prescription for chronic obstructive pulmonary disease:a systematic review with meta-analysis. Int J Environ Res Public Health 2018;15:E1830. 30149535 10.3390/ijerph15091830 PMC6165467

[18] Saeed S Antonacci DJ Bloch RM. Exercise, yoga, and meditation for depressive and anxiety disorders. Am Fam Physician 2010;81:981–6. 20387774

[19] Tsang H Fung K Chan A. Effect of a qigong exercise programme on elderly with depression. Int J Geriatr Psychiatry 2006;21:890–7. 16955451 10.1002/gps.1582

[20] Liu X Clark J Siskind D. A systematic review and meta-analysis of the effects of Qigong and Tai Chi for depressive symptoms. Complement Ther Med 2015;23:516–34. 26275645 10.1016/j.ctim.2015.05.001

